# Case of Cretinism

**Published:** 1893-03

**Authors:** Hugh Hagan

**Affiliations:** 211 Peachtree Street; Atlanta, Ga.


### "CASE OF CRETINISM."

ATLANTA, GA., February 27, 1893.

*To the Readers of The Atlanta Medical and Surgical Journal:*

In the February number of this JOURNAL I reported a case of Cretinism, which occurred in my practice. I stated as evidence of the rarity of this remarkable psychosis, the fact that a man as prominent as Dr. Spitzka had reported only three cases, and those not true types of cretinism .

I learn that since the publication of the report of this case several letters have been received by the editor of the JOURNAL, calling his attention to the fact, that they too had reported cases of cretinism, and would like to be quoted or given their proper place in the lists of the recorders of this disease.

Now by way of assuring any one who has been so fortunate as to record a case of cretinism in America, and who might have deemed himself ignored, I offer the following as evidence of my good will:

I would not for the world have any one think my recital of these four cases was meant to convey the idea that no other cases of cretinism had been recorded in the reports of American physicians, or that I was not cognizant of the existence of other recorded cases, or that I intentionally neglected to quote those who had recorded their cases, or that I hoped to be number two on the list of recorders of cretinism in America; but simply and without malice, mentioned the name of the author freshest in my mind, willing at the same time to render honor to whom honor was due. HUGH HAGAN, M. D.

211 *Peachtree Street.*

---

### PNEUMONIA.

℞. Ammonii muriatis, ʒj.

Extract glycyrrhizæ, ʒj.

Spts. ætheris sulph., ʒij.

Aquæ, ℥iv.

M. Sig.—A tablespoonful every two or three hours. (In advanced stages of pneumonia.)—*Waring.*

℞. Tinct. veratri viridis, minims xl.
Spts. ætheris nitrosi, ʒvj.
Liq. potassii vitratis, ʒivss.
Syr. zingiberis, ad ℥vj.

M. Sig.—A tablespoonful every three hours. (In the early stage.)—*Da Costa.*

℞. Potassi iodidi, ʒj.

Ammonii muriatis, ʒiss.

Mist. glycyrrhizæ co., ℥vj.

M. Sig.—A tablespoonful four times daily, to promote absorption, together with blisters to the chest.—*Da Costa.*

—*Medical World.*

